# “Actually, the psychological wounds are more difficult than physical injuries:” a qualitative analysis of the impacts of attacks on health on the personal and professional lives of health workers in the Syrian conflict

**DOI:** 10.1186/s13031-023-00546-5

**Published:** 2023-10-09

**Authors:** Aula Abbara, Diana Rayes, Hannah Tappis, Mohamed Hamze, Reham Wais, Hesham Alahmad, Naser Almhawish, Leonard Rubenstein, Rohini Haar

**Affiliations:** 1 Syrian American Medical Society, Washington, DC USA; 2 Department of Infectious Diseases, Imperial College, London, St Marys Hospital, Praed Street, London, W2 1NY UK; 3 Syria Public Health Network, London, UK; 4 Johns Hopkins Bloomberg School of Public Health, Baltimore, USA; 5 Syrian American Medical Society, Gaziantep, Turkey; 6 Assistance Coordination Unit, Gaziantep, Turkey; 7 School of Public Health, University of California, Berkeley, Berkeley, USA

**Keywords:** Syria, Conflict, Health workers, Moral injury, Moral distress, Attacks, Coping

## Abstract

**Introduction:**

Attacks on healthcare in armed conflict have far-reaching impacts on the personal and professional lives of health workers, as well as the communities they serve. Despite this, even in protracted conflicts such as in Syria, health workers may choose to stay despite repeated attacks on health facilities, resulting in compounded traumas. This research explores the intermediate and long-term impacts of such attacks on healthcare on the local health professionals who have lived through them with the aim of strengthening the evidence base around such impacts and better supporting them.

**Methods:**

We undertook purposive sampling of health workers in northwest and northeast Syria; we actively sought to interview non-physician and female health workers as these groups are often neglected in similar research. In-depth interviews (IDIs) were conducted in Arabic and transcribed into English for framework analysis. We used an a priori codebook to explore the short- and long-term impacts of attacks on the health workers and incorporated emergent themes as analysis progressed.

**Results:**

A total of 40 health workers who had experienced attacks between 2013 and 2020 participated in IDIs. 13 were female (32.5%). Various health cadres including doctors, nurses, midwives, pharmacists, students in healthcare and technicians were represented. They were mainly based in Idlib (39.5%), and Aleppo (37.5%) governorates. Themes emerged related to personal and professional impacts as well as coping mechanisms. The key themes include firstly the psychological harms, second the impacts of the nature of the attacks e.g. anticipatory stress related to the ‘double tap’ nature of attacks as well as opportunities related to coping mechanisms among health workers.

**Conclusion:**

Violence against healthcare in Syria has had profound and lasting impacts on the health workforce due to the relentless and intentional targeting of healthcare facilities. They not only face the challenges of providing care for a conflict-affected population but are also part of the community themselves. They also face ethical dilemmas in their work leading to moral distress and moral injury. Donors must support funding for psychosocial support for health workers in Syria and similar contexts; the focus must be on supporting and enhancing existing context-specific coping strategies.

**Supplementary Information:**

The online version contains supplementary material available at 10.1186/s13031-023-00546-5.

## Introduction

Violence against healthcare is increasingly scrutinised for its direct and indirect impacts on population health, on the health system and on health workers. Health workers in particular are vulnerable to direct attacks globally, often deliberately and without accountability [1]. Syria’s brutal conflict which began after peaceful uprisings in March 2011 were violently suppressed by the Syrian government, has seen frequent, violent and deliberate attacks on healthcare, which have thus far occurred with impunity [2]. Early in the conflict, the provision of medical care to those considered opposed to the government was criminalised, leading to the targeting, arrest and torture of many healthcare workers and attrition of the health work force [3]. From 2014 and 2015 onwards, violence on healthcare has primarily been shaped by airstrikes against health facilities and other medical units by the Syrian government and its allies, with various peaks in the violence in 2016 in Aleppo, 2017 in Al-Raqqa and 2019 in Idlib governorates [4]. These attacks, as well as the pressures of conflict have forced an exodus of health workers, leaving those who remain facing increased pressures from working in an under-resourced health system where targeting of healthcare persists.

A 2021 Physicians for Human rights analysis found that healthcare workers who were detained for providing medical care to protestors in Syria often suffered significantly more in detention compared to non-health workers [4]. The Syrian American Medical Society (SAMS), a global medical relief organisation which has provided medical and humanitarian relief in Syria since the start of the uprisings, has produced a series of reports which explore the impacts of such attacks. Their May 2022 report ‘A Heavy Price to Pay’ examines attacks on health in Syria between 2015 and 2021 noting the devastating impacts of such attacks which resulted in interruptions to healthcare and a high toll on civilians [5].

Despite the impacts of such attacks, many health workers have stayed, often citing a sense of duty and personal ethics as a key driver of this decision [6]. The conflict as well as additional stressors, has taken a devastating toll on the population and the health system, presenting greater challenges for the remaining health workers to manage. As of June 2022, PHR reports 945 health workers have been killed as a direct result of the conflict [4], and more than 318 have been injured, including 60 women [5], with the majority of attacks perpetrated by the Syrian government (76%) and its Russian allies (12%) [4]. However, the less tangible effects of the conflict including mental health, stagnant career prospects, lost income as well as the wider impacts on their families is harder to quantify. While attacks have tapered in recent years, their impacts continue to deeply affect health workers and the community [7].

Academic collaborations as well as humanitarian organisations and civil society organisations have sought to characterise attacks [6–8] and explore the impact of attacks on health workers in Syria and elsewhere, however few explore the medium- or longer-term impacts from the perspectives of those health workers who have experienced such attacks directly.

## Methods

### Aim and study design

The aim of this study was to qualitatively explore the impacts of attacks on healthcare in Syria on the personal and professional lives of local health workers and, where possible, how these have been mitigated.

### Study setting

**Fig. 1:**
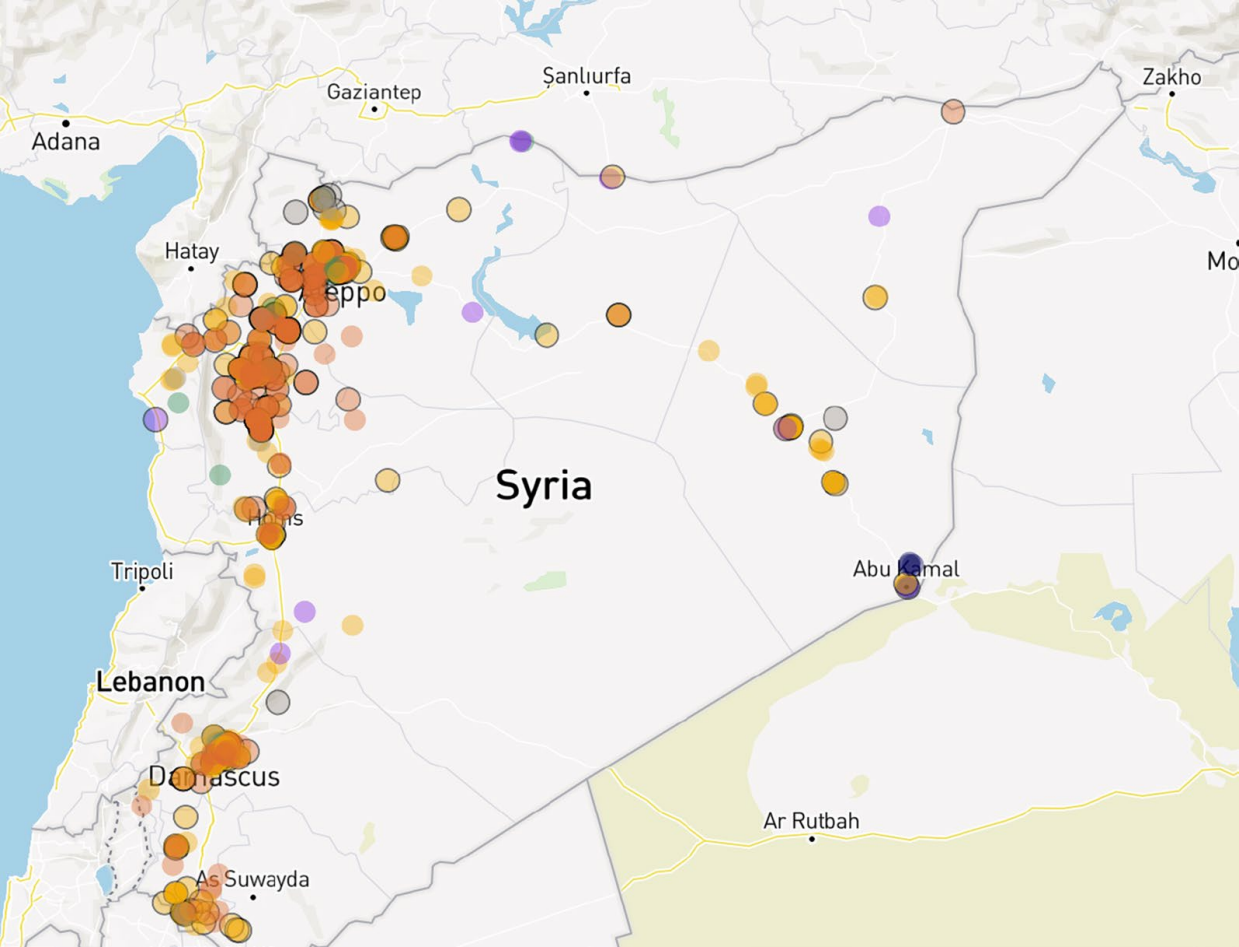
The distribution of attacks on healthcare in Syria between 2011 and 2021 [14]

The study was conducted predominantly in northwest Syria but includes some interviews from northeast Syria where ongoing conflict continues to affect healthcare workers as well as the population. We focus on these regions as they have faced the highest concentration of attacks. Northwest Syria is a geographical area which encompasses Idlib governorate and parts of Aleppo governorate; it shelters around 4.3 to 4.6 million people of whom more than 65% are IDPs and more than one million still reside in tented settlements [9, 10]. Around 60% of health facilities are fully functioning [11]. Northeast Syria contains around 2.65 million people [9, 12] (though the quoted figure can be as high as 4 million) of whom 23% are IDPs and covers the governorates of Hassakeh, Raqqa, Deir-Ez-Zor and parts of Aleppo governorates [8, 13]. Figure 1 shows the distribution of attacks on healthcare in Syria between 2011 and 2022 from Physicians for Human Rights [14].

### Study partners

The study was conducted in partnership with two humanitarian organisations who work on the humanitarian health response inside Syria: SAMS and the Assistance Coordination Unit (ACU). SAMS has provided health and humanitarian services since the onset of the conflict and has been a key provider of services in northwest Syria; it has faced more than 222 attacks on its health facilities and has documented such attacks since 2015 [5]. The ACU leads the EWARN (Early warning and response network) syndromic surveillance program for infectious diseases and is active in areas outside of government control in Syria [15].

### Qualitative methodology

We used a qualitative methodology to contextualise and understand health worker perceptions and mitigation strategies in relation to attacks on healthcare. The overarching research questions guiding interviews were: (1) What are health worker perceptions of the impact of violence on healthcare based on their experiences and knowledge of attacks? and (2) How did violence against healthcare impact and shape the personal and professional experiences of health workers operating in this context? These questions followed an initial discussion where health workers were asked to cite specific events or experiences with attacks on healthcare. The goal of this was to facilitate recollection of these events.

## Sampling

We used a purposive sampling approach to recruit Syrian health workers using an inclusive definition of a health worker to include those who are in administrative roles (e.g., managers, receptionists) as well as clinical roles. This provided greater depth than previous studies and allowed for a deeper understanding of the impacts on the health workforce. We note that prior qualitative research conducted with Syrian health workers often focused on physicians and women were poorly represented due to challenges to access for participation. We therefore purposely included non-physician and female health workers. We drew on the network of SAMS, ACU and other humanitarian organisations which work in northwest and northeast Syria.

Inclusion criteria were defined as follows: participants had to be at least 18 years old and able to consent; fluent in Arabic or English; have online access to Zoom or a telephone, identify as a health worker or managerial staff responsible for the coordination of humanitarian and health relief efforts, and be currently working in Syria. A snowballing approach was then used to identify other individuals who were interested in participating in the study, and particularly, to invite participation from female and non-physician health workers.

### Data collection

We conducted open-ended, semi-structured in-depth interviews (IDIs) with Syrian health workers to explore their experience of attacks on health care, particularly the effects of attacks in the medium and longer term. We explored what mitigating factors they have used if at all. The questionnaire is provided as Additional file 1. All interviews were conducted by experienced bilingual (Syrian Arabic/English) investigators via Zoom as interviews were set to commence during the beginning of the COVID-19 pandemic and travel was not possible. The majority of interviews were audio-recorded for transcription of interviews and maintaining accuracy of information shared, with a few exceptions where handwritten notes were taken based on participant preference. Interviews were transcribed in Arabic and translated to English by native Arabic speakers for coding and analysis.

### Data analysis

We analysed the data using the Framework Method developed by Gale et al. 2013, which is commonly used to analyse health research using a multi-disciplinary approach [16]. One key distinction of this approach is that it permits both inductive and deductive approaches to thematic analysis. We followed the steps proposed by the Framework Method including familiarisation with the interviews, initial coding, developing an analytical framework (set of codes that are mutually agreed upon), and charting data into a framework matrix. Interviews were both inductively and deductively coded using Dedoose 9.0 by two individual coders (DR, AR) and compared and discussed for discrepancies. A third coder also supported the analysis (HT). Coding and subsequent interview results were discussed iteratively among the qualitative research team on a regular basis, identifying potential themes of interest and areas for further exploration. Themes were developed based on the perceptions of healthcare workers regarding the various impacts of violence against healthcare. We identified key themes, patterns, and correlations among IDI responses. Emerging findings from the qualitative data were also discussed with the larger study group involving the quantitative analysis in order to ensure consistency and explore any incongruous data.

### Ethical considerations

UC Berkeley (UCB) provided ethical approval for this study (Protocol ID: 2020-03-13069). Because no formal body exists that could serve as a local review board, UCB consulted with the Idlib Health Directorate and other local leadership before administration of the qualitative and quantitative components of this work, to obtain their input, feedback, and approval. All study participants undertook a verbal informed consent process in Arabic.

In order to protect respondent confidentiality and protect them from possible recriminations from the Syrian government, minimal details (age, gender, profession, governorate location) were recorded about the characteristics of each respondent. Data were stored on a single, password protected computer and, where transfers were made, only encrypted servers were used. Participants could withdraw at any time before analysis commenced.

## Results

Between June 2021 and November 2021, a total of 40 health workers who had experienced attacks between 2013 and 2020 participated in IDIs. Participants included 27 males (67.5%) and 13 females (32.5%) and represented a variety of health cadres, including nurses (32.5%), physicians (27.5%), health administrators (15%), technicians (laboratory, surgical assistant, or anaesthetic) (12.5%), dentists or pharmacists (7.5%), and medical students (5%). These are summarised in Table 1.

**Table 1:** Participant characteristics

Total participants	40	
Gender	*Female*	13
	*Male*	27
Location	*Aleppo*	12
	*Ghouta*	3
	*Hama*	2
	*Idlib*	18
	Kafranbel	1
	Raqqa	4
Profession	Nurse	10
	Technician	6
	Student	3
	Administrator	4
	Physician, Dentist, Surgeon	9
	First Aid Worker	4
	Pharmacist	2
	Midwife	1
	Psychologist	1
Age Range	20–29	14
	30–39	12
	40–49	2
	50–59	5
	≥ *60*	2
	*Unknown/Not shared*	5

### Description of context

Participants were mainly based in Idlib (39.5%) and Aleppo (37.5%) governorates with fewer from Raqqa (10%), Eastern Ghouta (7.5%), and Hama (5%) governorates. A wide variety of facilities were mentioned by participants as targeted centres including from general hospitals (46%), surgical centres (33%), primary care centres (13%) and specialty hospitals (8%). An attack on Ariha Surgical Hospital in Idlib, Syria in 2019 had left a strong impression on many participants from Idlib governorate, as they dealt with the direct and indirect repercussions of the attack. All participants reported direct experiences with violence against healthcare, such as being victims of bombing of facilities, the targeting of ambulances, and arrest of health workers; most reported two or more direct experiences.

### Key themes

The main emerging themes were as follows: (1) psychological harms; (2) impact of the nature of attacks on healthcare; (3) coping mechanisms.

### Psychological harms

A prominent emerging theme related to internal tension between the personal and professional impacts on health workers and the effects on their socio-emotional well-being; these often manifested as tensions between their personal needs and their sense of duty to patients and the community. They reported concern from them, and their families related to the physical risk of severe injury or death as well as the psychological impacts which include burnout, exhaustion, and symptoms of stress. This was exacerbated by working in a system which was severely understaffed relating to the forced exodus of other health workers. Despite this, participants spoke of their commitment to remain.

“But for me, I absolutely refuse to leave Syria. In my opinion we must do anything that can support the Syrian revolution, God willing.” *(Participant 3, Administrator)*

“I was more determined to serve patients in these poor conditions. I saw my dearest person, the administrative director of the hospital, who died because of the targeting, which affected me psychologically, but there are priorities; serving people; beneficiaries and patients.”* (Participant 2, Nurse)*

For those left behind, there was the additional professional sense of obligation to fill gaps of health workers who were forced to leave:

“Honestly, with regard to the health sector, there is a huge gap of staff. Many of the doctors who stayed in Syria died, and those who decided to emigrate have emigrated, and what remains is the very few.” *(Participant 20, Nurse)*

As in other conflict settings, health workers themselves also faced the same challenges and lived experience of others during the conflict. However, due to the targeting of health facilities, they were often at greater risk. One participant noted:

“I always imagine myself leaving, leaving home to work, saying goodbye to my kids and going to work; I might not come back to see them again. The thing is that always, … I might die, or they might die due to bombardment or shelling. It is always on my mind. The magnitude of tension in which I live … I don’t know.” *(Participant 4, Medical Technician)*

The mental health of health workers came across strongly as a key topic, something which is important given its generally stigmatised nature in Syria. Interviewers noted hesitation or embarrassment among health workers who appeared reluctant to admit experiencing symptoms related to mental health concerns. However, almost all participants alluded to it, whether directly or indirectly. Participants noted,

“Actually, the psychological wounds are more difficult than physical injuries.” *(Participant 7, Physician)*

“We started feeling very, very, very bad psychological pain that you cannot imagine which badly affected our personal lives. When we were back home and trying to live our personal and normal lives with family and friends, we could not do that because we were living in a constant state of stress” *(Participant 30, Surgeon)*

Some participants captured the severe symptoms and reactions which many of the IDIs highlighted. A total of 9 participants mentioned the attack on Ariha Hospital, including the killing of a hospital administrator which left a huge impact on the staff he left behind. One participant who had survived the targeting of Ariha Hospital in Idlib expressed significant symptoms of stress related to the conflict and targeting:

“I get this anxiety whenever I remember the time we were targeted; just like now. I am telling you about it while my hands are shaking. I feel stressed, provoked, wanting to retreat to myself, and stay alone more than before.” *(Participant 4, Medical Technician)*

“But I did witness the targeting of Ariha Hospital, all of it, everything, I witnessed it all, I mean, we reached a point when we were just expecting it to happen…, when we had enough and prayed for the air force to attack us and to die because we were experiencing the fear of death over and over again, we were saying, “I want this attack to hit me, I had enough, I want to die, enough, I got so afraid that I was not able to endure anymore, I couldn’t endure all those attacks and bombardment.” *(Participant 4, Medical Technician)*

Participants noted personal impacts including multiple experiences of displacement, leading to inconsistency in employment, lack of livelihoods, and psychological and physical strain on health worker families. In the Ariha hospital, most health workers were so shaken by the attack, they refused to return to work:

“But the staff said that if there is a hospital re-established in Al-Ariha, we will not come back to work. The staff were not in a good psychological state after the attack. Even those who used to work there completely refused to return.” *(Participant 3, Administrator)*

Female health workers faced some challenges which male health workers did not; these included, for example, greater concern from their families around security and the redistributing of family responsibilities such as childcare. Families sometimes requested them not to go to work, particularly at nightfall or when violence had escalated in the surrounding community. Female health workers also faced additional pressures as a minority in the workforce, despite a high demand for services such as nursing and midwifery:

“When a hospital was bombed nearby, and closed, … all people were displaced to us, so more pressure was on inspections, more pressure for women, more pressure for hospitals, because all of these hospitals were closed” *(Participant 20, Nurse)*

The personal and ethical tensions were reflected in some of the difficult decisions and circumstances which health workers found themselves needing to make. For example, one participant notes:

“I have to still focus, as there is human life in my hands. However, my focus gets distracted. You probably can't see that I am focused and distracted at the same time. Aaah, but this is what happens with us, that I have a patient in my hand, neither I nor the surgeon who stands with me, nor the surgeon’s assistant, nor nurses who are here can leave him. If there was an attack and we could carry this patient out and leave with him, honestly, we would do it to be safe with him.” *(Participant 16, Medical Technician)*

A small number of participants reported feeling desensitised to the impact of attacks or getting used to a "new normal." This means of self-preservation may be useful in the time of danger, however, may have lasting consequences for the health workers and their families without the required support.

“The first three years may be difficult but then we have adapted to war, it becomes normal for us. On one day, there was constant bombing by helicopters and war-planes for the whole day from morning to evening. It becomes a routine. After the amount of bombings, we would see, it would become normal. Sometimes we bury tens of martyrs in one day. This become normal and our psychological state is no longer affected by these things.'' *(Participant 3, Administrator)*

### Impact of the nature of attacks on healthcare and conflict violence

An important factor which affected the health workers was the distinct nature of the attacks on health in Syria. This included the ‘double-tap’ method of attacks favoured by the Syrian government and its Russian allies where the first attack is followed on by another moment or hours later. These repeated attacks create what many participants described to be anticipatory stress of further attacks in the immediate aftermath of the first when they were rapidly working to manage and evacuate patients while attempting to avoid further casualties. A participant described the experience of waiting for these inevitable second attacks:

“These were some of the hardest moments. You had a patient so you cannot leave the hospital, but the war planes were bombing the area and the ambulance could not reach you to take the injured person. These were very difficult moments.”* (Participant 3, Administrator)*

Participants described the pressure which health workers faced in taking up multiple roles including immediate medical relief, ensuring oxygen supplies for neonates in incubators and supporting evacuations in anticipation of further attacks:

“[We were] working despite knowledge that an attack was imminent. We have been expecting the attack at any moment. But we had to continue working. We cannot stop. There were injuries, patients, and scheduled operations. We had just tried to postpone cold [non-emergency] operations and focus as is possible on urgent tasks, especially the ones caused by the bombardment. We were at the front line by that time. We had been the first defence line for cases and injuries from bombardments against civilians.” *(Participant 4, Medical Technician)*

All the participants reported at least two exposures to attacks on healthcare, perhaps related to the ‘double-tap’ nature of attacks on health and repeat attacks on health facilities. A participant said:

“Throughout the war, we learned that if one attack happens, then another attack will happen in the same place, where the most wounded could occur. This is the lesson we learned from the attacks that were happening.” *(Participant 9, Nurse)*

Another participant spoke to the disorienting nature of these attacks:

“Any attack can cause disorder in the entire hospital. We cannot recognize our colleagues and we didn’t know who died or [was] still alive, actually we need 15 minutes as a minimum to understand the situation and determine the extent of the damage. You know after the attack you may lose some of your senses, such as hearing or vision for a while, something hard to describe*.” (Participant 7, Physician)*

More broadly, the conflict-related violence across the community resulted in a complex intersectionality for many health workers. As neighbours and community members they experienced everything that happened in the community—food and fuel shortages, civilian attacks, health issues and more. As health workers, they had to both treat the wounded and sick and manage the direct violence they were experiencing, both heightened when conflict escalated. These roles complicated both the day-to-day experiences of health workers trying to balance family life with professional obligations, but also the wider view of what health workers are to the community.

“One time I was operating, and the western side of [my city] was bombarded. My house was on the western side.… My wife and my little children were there. I just wanted to turn on my phone to check if they were alive or dead. This happens all the time.” *(Participant 34, Physician)*

### Coping mechanisms

Despite the challenges faced by the health workers, there were examples of active and passive coping including resilience. In psychosocial research, coping can take behavioural, emotional, or cognitive dimensions to manage stressful events [17], all noted to be components of coping among our participants. Participants also noted both passive and active coping strategies in their day-to-day work. A strong sense of solidarity was in evidence among the participants, particularly among those who had survived the Ariha bombings. The mental health needs of the health workers (as well as the broader population) were great, however stigma related to admitting the need for psychological support or experiencing symptoms and the lack of availability of needed services left this as an important gap. In addition, participants reported what could be described as negative coping mechanisms including the use of sedatives or engaging in dark humour (Participants 1, 4, 8). However, participants spoke of the importance of bonding or talking with colleagues as a means of providing emotional and social support with those who could understand the pressures they faced (Participants 9, 14, 16, 23, 25, 27).

## Discussion

This study, which explores the compounded impacts of violence against healthcare in Syria on the health workforce, contributes complex and nuanced personal perspectives which explore this increasingly important issue. Attacks on healthcare have deep, complex, and long-lasting impacts on health workers, some of which are highlighted in this study. Syria has seen the relentless and often deliberate targeting of healthcare—a violation of international humanitarian law and a precedent which is now increasingly seen in other conflicts including Ukraine and Sudan [18, 19]. As such, healthcare workers who remain in Syria have faced a relentless conflict but also the pressure to care for a population which faces an increasing burden of disease related to the armed conflict, the breakdown of health infrastructure and under-resourcing [10]. Health workers are both community members and humanitarian responders in increasingly complex social structures resulting from a changing society due to the protracted armed conflict. This has been particularly stark in the aftermath of the February 2023 earthquakes which affected northwest Syria where the health workers were both direct victims and responders [10].

The pressures on healthcare workers who remain in Syria and who have experienced both direct and vicarious traumas related to both attacks on healthcare and the effects of living through protracted conflict were highlighted by all participants. While there is literature relating to the impact of the armed conflict among the general population of Syria [10, 20] exploring the challenges which health workers themselves face there are more limited. In these few instances, studies found that health workers subjected to repeated attacks resulted in psychological and emotional repercussions among survivors [6, 21, 22]. This finding is given that the nature of the attacks by Syrian government forces and their Russian allies contributes to these outcomes. In particular, the ‘double-tap’ attacks which have been used as a tactic, particularly by Russian forces, appear to amplify the fear and stress which healthcare workers experience in the immediate aftermath of an initial attack on a health facility. We describe this experience as ‘anticipatory stress’. This has also been used more recently by Russia in Ukraine [23, 24] where rescue crews arriving in the aftermath of an attack have been targeted.

Our findings are consistent with studies of the impact of attacks on health workers in other conflict settings with some similarities in the experience noted. For example, Elnakib et al. describe the impacts on Yemeni health workers who report disempowerment, distress and burnout in the face of such attacks while also reporting a duty to serve and engaging with coping mechanisms [25]. The latter included turning to faith and demonstrating resourcefulness and innovations in their professional work. Sousa and Hagopian describe how Israel’s military occupation of the West Bank affected Palestinian medics. The experience of harassment and violence by Israeli soldiers created great stress on medics, and they suffered moral injury from not being able to perform their jobs in serving people in need. Their coping mechanisms derived from their roots in the community and solidarity with the community and their colleagues [26]. In Myanmar, too, the rootedness of health workers in communities became a major means both of ensuring access to health care in the face of persistent violence and to cope with violence when it was inflicted on them [27].

Witter et al. explored coping mechanisms among health workers in Uganda, Sierra Leone, Zimbabwe, and Cambodia; such contexts had experienced different shocks including conflicts and prolonged political-economic crises [28]. They note some similarities to our findings relating to the challenging working conditions, including the fear of and risk of death resulting, particularly affecting their psychosocial well-being [28]. Recent surveys from northeast Nigeria and South Sudan noted a range of threats and attacks to healthcare workers resulting in psychological impacts as well as wider impacts on the respective health systems and access to healthcare [29, 30].

Violence against healthcare workers became more prominent during the COVID-19 pandemic, leading to calls for frontline health workers to be protected [1]. Though such attacks against healthcare in non-conflict affected areas could differ in their range and impact, these acts of violence were rightly condemned. This also led to calls to consider such violence against healthcare workers to be a political problem and a public health issue by Kuhlmann and colleagues [1]. They highlight this as a problem during COVID-19 such they call for policies which focus on preparing and protected health workers and establishing monitoring and reporting systems on such violence [31]. Beillizi et al. also emphasise the issue of attacks on health outside of conflict settings [32], stating that up to 38% of healthcare provisions were subject to physical violence at some point in their lives. It is however important to emphasise that though some similarities exist to the threat of direct violence against healthcare workers outside of conflict settings, there are particular conflict related factors, particularly where attacks on healthcare are used strategically against healthcare [33].

The nature of the attacks on health, where healthcare workers describe trying to respond while also ensuring that they and their colleagues and families are safe also presents ethical dilemmas for the healthcare workers. This has been explored in previous research by Rubenstein [34], Singh et al. [6] and Kallstrom et al. [35] who note the multiple, intersecting personal and organisational challenges which healthcare workers in northwest Syria face. In particular, the inner conflict and distress experienced by the healthcare workers while they experience both the extreme distress and compulsion to remain to serve despite the risks is mirrored in our work. Kallstrom et al. also refer to both intrinsic e.g. humanitarian principles, medical ethics and extrinsic reasons e.g. ideological, patriotic, political and religious as motivators to continue working in Syria, despite the dire circumstances [35].

Our findings reinforce the need to further explore aspects of ‘moral distress/ moral injury,’ something which is increasingly discussed in the wake of the COVID-19 pandemic where life-saving resources have been constrained, even outside of conflict settings [36, 37]. There has been little primary exploration of moral distress in other conflict settings though it continues to be recognised as important in current armed conflicts and their aftermath including in Ukraine, Tigray and Afghanistan [38]. However, as Singh et al. note, empowering healthcare workers and exploring the ethical humanitarian challenges or providing decision making support for the more challenging ethical decisions which they face can support a reduction in moral distress [6]. Broussard et al. explore this at the organisational level for humanitarian organisations where they have ethical and humanitarian duties to uphold and a duty of care to the healthcare workers under their jurisdiction [39].

Despite the challenges faced, those who remain in opposition-controlled Syria do so for a sense of duty to their communities but have needed to develop different coping mechanisms to survive in these abnormal circumstances. They also described some practical adaptations including fortifications to health facilities or moving them underground to minimise the impacts of attacks [40]. There were also other risk mitigators put in place which included choices around the locations of health facilities. However, for the health workers, the recurrent nature of the attacks and the intentionality with which attacks occurred continued to take a toll. Participants also described a range of personal coping mechanisms—behavioural, cognitive, emotional, and religious that played a role. Broadly, these coping mechanisms can be divided into ‘active’ coping where problem solving may be employed or ‘passive’ coping. The latter describes a sense of hopelessness or resignation to the circumstances [41]. Additionally, in some of the interviews, there was an absence of coping mechanisms, highlighting the needs for more psychosocial support appropriate to health workers in such settings. In general, ‘passive’ coping which may include emotional avoidance or passive religious coping may increase anxiety-depression in the long term despite reducing emotional reactivity in the short term [42]. Such coping strategies were also identified among health workers in Yemen [25]. Among our interviews, it is notable that faith played an important part with most engaging with it as a means of ‘active’ coping. Some Syrian health workers, as in other settings where they face attacks may channel their efforts into ‘resistance’ or advocacy to ameliorate the sense of hopelessness they may feel. Much of the literature on coping in the face of armed conflict focuses on civilians rather than on health workers suggesting that further exploration of this is required, given potential differences in needs and responses [43].

How such attacks and their impacts on healthcare workers can be mitigated remains an important issue. Though attacks on healthcare facilities and healthcare workers are widespread, there is little accountability to the perpetrators of such attacks. Initiatives such as RIAH (Researching the Impacts of Attacks on Health [44]) seek to foster better understanding of the impacts of attacks on health with a view to enabling mitigation measures and informing accountability. These are, however, long-term goals. In the short term, there must be cessation of such attacks which have such profound impacts on healthcare workers and the communities they serve. For healthcare workers in opposition-controlled Syria who contend not only with the attacks on healthcare but living through protracted conflict and the February 2023 earthquakes, there is an urgent need to support and protect them particularly from the psychological harms which have long term consequences. This includes organisational structures, processes, training, supports and safe reporting mechanisms to help their staffs cope with the extreme dangers, stresses, and moral injuring from working in settings where they are subjected to violence, including gender-specific needs; and donor commitment to fund psychosocial interventions and support, tailored to the needs of health workers [34]. Failure to do so impacts not only the healthcare workers themselves but also the wider communities which rely on them.

## Limitations

Our use of convenience and snowballing sampling may have contributed to a bias as to who was interviewed, and we note that the majority of interviewees resided in northwest Syria rather than in other parts of the country. However, given that this area has suffered among the most protracted and ongoing attacks on healthcare, this should enrich rather than restrict the study findings. Despite extensive efforts to recruit female health workers, we were unable to achieve equality in numbers though in this study, we succeeded in including a greater number compared to other qualitative studies conducted in the region. This likely reflects the demographic makeup of health workers and challenges to access. However, gendered issues in relation to attacks on health require further exploration [45]. Additionally, our research has focused on the perspectives of those who remain in northwest Syria and not those who left as a result of the attacks on health and insecurity. They may describe similar perspectives or differences; for example, they may harbour guilt for leaving.

## Conclusions

In this study, we shed light on the impact that both the extent and nature of attacks on healthcare in Syria have had on healthcare workers. Such findings have relevance in other contexts including in Ukraine where similar tactics have been used by Russia as well as the more recent conflicts in Sudan where healthcare has been targeted since early in the escalation. We note that mitigation and coping strategies must be strengthened and that support with the challenging ethical decision making required of healthcare workers in such settings is essential. Lastly, the more gendered impacts on female health workers and medical students need further exploration, something which is urgent given the role which women have in their communities and in healthcare.

### Supplementary Information

**Additional file 1.** Semi-Structured Interview Guide.

## Data Availability

The datasets generated and/or analysed during the current study are not publicly available due to the sensitive nature of the individual interviews but are available from the corresponding author on reasonable request.

## Abbreviations

ACU: Assistance Coordination Unit
EWARN: Early warning and response network
IDIs: In-depth interviews
IDPs: Internally displaced people
PHR: Physicians for Human Rights
R2HC: Research for health in humanitarian crises
SAMS: Syrian American Medical Society
UCB: University of California, Berkeley
