# Supplementary material for: “Actually, the psychological wounds are more difficult than physical injuries:” a qualitative analysis of the impacts of attacks on health on the personal and professional lives of health workers in the Syrian conflict
Source: Confl Health. 2023 Oct 9;17:48. doi: 10.1186/s13031-023-00546-5 (PMC10561459; doi:10.1186/s13031-023-00546-5)
Supplement: Supplementary file 1 — Additional file 1. Semi-Structured Interview Guide. [file 13031_2023_546_MOESM1_ESM.docx]

**Semi-Structured Interview Guide – KEY INFORMANT INTERVIEWS (v.2)**

| ***STUDY TITLE: Impact of violence against healthcare in Syria*** | | | |
| --- | --- | --- | --- |
| *Interview Type* | *Key Informant/Individual* | | |
| *Participant(s) ID* | *_________________* | | |
| *Interviewer ID* | *_________________* | | |
| *Interview Date* | *______ (DD) / ______ (MM) / ___________ (YYYY)* | | |
| *Interview Time* | *______ (Hour) / ______ (Minute) AM / PM* | | |
| *Interview Duration* | *______ (Minutes)* | | |
| *Interview Location* |  | | |
| *Interview Language* | *___Arabic ___English ___Other______________* | | |
| *Data Recording* | *___Audio-recorded ____Hand-written notes* | | |
| ***PARTICIPANT INFORMATION*** | | | |
| *Participant Age* |  | | |
| *Participant Sex (Observed)* | *___Female ___Male* | | |
| ***COMPLETE AFTER INTERVIEW*** | | | |
| *Participant Questions and Feedback* |  | | |
| *Interviewer Observations and Feedback* |  | | |
| *Interviewer Questions about Interview* |  | | |
| *Problems or Adverse Events* | *Description of Problem:* | *Steps Taken:* | *Resolution:* |
| *Field Supervisor Review (Check when complete)* | *___ Data collection form*  *___ Audio-transcription/ Hand-written notes*  *___ Informed consent form signed by interviewer* | | |

*Note: These are illustrative guides that will be finalized and pre-tested with our local research partners.*

*Thank you for agreeing to participate in this study. One of our study objectives is to understand the indirect and direct impacts of different types of violence against healthcare, such as attacks on hospitals, or being threatened as a health provider, on the delivery of health services and on community health outcomes inside Syria. We may also ask you to recall specific incidents in the past in order to provide a broader understanding of the impacts of these incidents.*

**PART 1. INTRODUCTION**

*I will start by asking you a few questions about yourself and your work.*

1. Can you tell me a little bit about yourself?

*Probes:*

- - *What is your healthcare specialty?*
  - *What are your main responsibilities?*
  - *How long have you been working in this position/ organization?*

1. Can you tell me about your previous work experience?

*Probes:*

- - *What kinds of services have you provided?*
  - *Which populations do you work with?*
  - *Where has your work primarily been (geographical area, public/private sector)?*

**PART 2. PERCEPTIONS AND KNOWLEDGE OF IMPACTS OF VIOLENCE ON HEALTHCARE**

*I will now ask you about the direct and indirect impacts of different types of violence on health service delivery/provision in your hospitals, facilities, clinics, etc.*

***SERVICE DELIVERY***

1. How has violence against healthcare impacted the delivery of health services in your community? (Includes impact on health infrastructure, health services, health workers, etc. )

*Probes:*

- *Impact on health infrastructure: Tell me about any hospitals, clinics, or programs that have been affected because of violence.*
- *Impact on health services: How have the types and scope of services changed because of violence? (quality and quantity)*
- *Impact on health workers: How has the violence affected the retention of health workers*

***COMMUNITY HEALTH OUTCOMES***

1. How has violence against healthcare impacted health outcomes in your community?

*Probes:*

- *Tell me about any health outcomes that have emerged because of attacks on health*
- *Why have these issues emerged?*
- *How have health workers addressed/coped with this?*

1. What new or changing health outcomes have emerged as a result of violence against healthcare?

*Probes:*

- *Types of health outcomes*
- *Numbers of patients/prevalence of disease*
- *Volume of health worker needs*

**PART 3: SPECIFIC INCIDENTS**

*Now, I will reference specific events or incidents based on the date or geographical location that you personally responded to/survived/witnessed. I may ask you to expand on what happened and how you all think or believe this impacted health service delivery in your geographical area/community.*

1. Can you share concrete examples of violence on healthcare that you all experienced (individually, as a group) during [this time, this place]?

*Probes:*

- - *Where were you?*
  - *What was your role in responding to the attack?*
  - *What was the impact on the local community?*
  - *Did anyone have a different experience?*

1. Can you please describe your memory of the...(insert incident here, i.e. siege on Aleppo, Idlib, particular time [2016], etc.)

*Probes:*

- - *Where were you?*
  - *What was your role in responding to the attack?*
  - *What was the impact on the local community?*
  - *Did anyone have a different experience?*

**PART 3. PROFESSIONAL AND PERSONAL RESPONSE TO VIOLENCE AGAINST HEALTHCARE**

*Now I want to ask you about your individual experiences with delivering health services in contexts of violence against healthcare. Since this project is focused on the impact of violence and attacks on healthcare inside Syria on health care providers, we want to learn more about how this specific type of violence may have affected your professional and personal experience.*

***PROFESSIONAL***

1. Can you tell me more about how violence or the risk of violence against healthcare affects you professionally?

*Probes:*

- - *What is it like to work in conditions of violence against healthcare?*
  - *What impact did this violence have on your ability to work?*
  - *How do these conditions affect how you provide patient care?*
  - *How do you balance your own risk and safety with your job responsibilities?*
  - *Did it affect you individually?*

1. Are there any particular difficulties you faced in your work as a woman [or man] in violent contexts?

*Probes:*

- - *Experiences of discrimination, harassment, or gender-based violence?*
  - *How do you balance your own risk and safety with that of your family and loved ones?*
  - *Can you tell me about a time you felt unsafe or uncomfortable because of the violence against healthcare?*
  - *How did this impact your work and willingness to stay in Syria/or to leave Syria?*

***PERSONAL IMPACT***

1. Can you tell me more about how violence or the risk of violence in your work affects you personally?

*Probes:*

- - *What is it like to live in these conditions?*
  - *What impact did violence against healthcare have on your motivation/willingness to stay in Syria?*
  - *How do these conditions affect you personally? (in the past, now, and in the future)*
  - *How do you balance your own risk and safety with that of your family and loved ones?*
  - *Did it affect you individually?*

1. Can you tell me about a time you felt unsafe or uncomfortable with because of the violent conditions in which you had to work or live?

*Probes:*

- - *How did this impact your work and willingness to stay?*

**CLOSING**

1. Is there anything else you would like to say, or you feel we should know to understand the direct and indirect impacts of attacks on health in [LOCATION]?
2. Do you have any questions for us?
3. Are there any other individuals you think we should speak with to learn about their experiences?
4. Can you recommend someone else at your organization that you think we should speak with?
5. Would you be willing to be contacted for a follow-up interview?

***Thank you very much for your time.***
